# Prevalence of Anti-JC Virus (JCV) Antibodies in the Multiple Sclerosis (MS) Population in Cyprus: A Retrospective Study

**DOI:** 10.1155/2019/3741260

**Published:** 2019-08-14

**Authors:** Sakis Lambrianides, Christiana A. Demetriou, Andis Tillyris, Elena Kkolou, Eftychia Gaglia, Eleni Agkastinioti, Eleni Leonidou, Yiolanda-Panayiota Christou, Savvas S. Papacostas, Kleopas A. Kleopa, Theodoros Kyriakides, Marios Pantzaris

**Affiliations:** ^1^Neurology Clinics, The Cyprus Institute of Neurology and Genetics, Nicosia, Cyprus; ^2^Department of Primary Care and Population Health, University of Nicosia Medical School, Nicosia, Cyprus

## Abstract

**Background and Purpose:**

Progressive multifocal leukoencephalopathy (PML) is a debilitating disease of the central nervous system caused by the ubiquitous polyomavirus JC (JCV) in immunocompromised hosts. In recent years, a new subpopulation of patients at risk for PML has emerged, due to the growing use of immunomodulatory or immunosuppressive therapies in autoimmune diseases such as multiple sclerosis (MS). The anti-JCV antibody index is used as a stratification tool in assessing the risk of developing PML. The objective of this study was to retrospectively describe the prevalence of anti-JCV antibodies in the MS population in Cyprus.

**Methods:**

We retrospectively collected the demographics of 214 MS patients in Cyprus who were screened for anti-JCV antibodies using the STRATIFY JCV™ assay between September 2011 and June 2018. Logistic regression analysis was used to examine the effect of demographic variables on seropositivity, and bivariate tests were used to assess the association between demographic characteristics and JCV AI index.

**Results:**

A total of 214 MS patients in Cyprus were tested. Overall anti-JCV antibody prevalence was 45.8% (95% confidence interval 37.2%–55.8%). We could not establish a significant association between seropositivity and increasing age or sex. In the subgroup analysis of natalizumab-treated patients, the annual seroconversion rate was 4.5%.

**Conclusions:**

Overall seroprevalence of anti-JCV antibodies in MS patients in Cyprus using the STRATIFY JCV assay was lower than the worldwide reported mean. Although previously reported, in our study, the anti-JCV antibody seropositivity was not associated with increasing age or sex.

## 1. Introduction

Progressive multifocal leukoencephalopathy (PML) is a devastating disease of the central nervous system, which was first described in patients with hematological malignancies in 1958 [1]. In 1971, the polyomavirus JC (JCV) was identified as the etiological agent [2].

JCV is a ubiquitous virus, with the anti-JCV antibody seroprevalence ranging from 39% to 91% depending on the sample size, the methodology of the assay, and the demographics of the population studied [3, 4]. JCV is highly specific to humans. There is only one major serotype, but there are at least seven major genotypes [5].

The mechanisms of JCV infection in humans are not completely understood, but it is assumed that the virus causes a subclinical infection and remains at a latent state in healthy subjects [6]. However, in the immunocompromised host, such as in patients with HIV or lymphoid malignancies, JCV reactivation can cause a lytic destruction of oligodendrocytes, resulting in PML [7].

The JCV viral particles are thought to be either ingested or inhaled and initially infect the endothelial cells of the kidney, establishing a persistent or latent subclinical infection. At one point, JCV can escape into the peripheral circulation and infect cells of haematopoietic lineage, such as B cells. Using haematogenous routes, JCV may then enter the central nervous system causing a lytic infection of the oligodendrocytes [6]. JCV can also be detected in reactive astrocytes as well as macrophages. The most prominent feature of the JCV infection in the brain is demyelination [8].

The largest population at risk for PML is the HIV+ patients. Although the introduction of combined antiretroviral therapy led to a significant decrease of PML incidence, about 80% of 9675 PML cases in the United States between 1998 and 2005 were attributed to HIV. The second largest population, consisting of about 10% of PML cases in the same period, was patients with hematological malignancies [9].

In recent years, a new population at risk for developing PML has emerged due to the increasing use of immunosuppressive and immunomodulatory treatments for autoimmune diseases [10]. While a significant number of drugs are known to cause PML [11], natalizumab, a monoclonal antibody used in the treatment of relapsing-remitting multiple sclerosis and of moderate-to-severe Crohn's disease, is associated with a particularly high risk [12]. The risk factors of developing natalizumab-associated PML are increased duration of natalizumab treatment, prior use of immunosuppressants, and a high anti-JC virus antibody index [13].

In the largest to-date multinational epidemiological study (JEMS study) of the anti-JCV antibody seropositivity in MS with a total of 7724 MS patients from 10 countries, the overall anti-JCV antibody prevalence was 57.1%. Prevalence increased with age and was lower in females. While the prevalence differed by country, no apparent geographical pattern could be elicited [14]. A recent systematic review of the worldwide prevalence of JCV antibody in MS and neuromyelitis optica patients, interestingly, also found prevalence to be 57.1% [15].

The primary goal of the current study is to describe the prevalence of anti-JCV antibodies in MS patients in Cyprus.

## 2. Methods

The study was approved by the Cyprus National Bioethics Committee.

We retrospectively assessed the database of MS patients who were screened for anti-JCV antibody in the Cyprus Institute of Neurology and Genetics (CING) in Cyprus during the period between September 2011 and June 2018. The CING is a tertiary referral centre for MS in Cyprus, where the majority of MS patients in the island are followed up.

Anti-JCV antibody testing was performed using the two-step enzyme-linked immunosorbent assay (ELISA), STRATIFY JCV™, which is considered the gold standard [16]. Demographics (age and gender) and antibody index were also evaluated. Anti-JCV antibody prevalence was estimated as the number of patients with detectable anti-JCV antibody as a percentage of all patients tested.

Descriptive statistics were calculated for the whole cohort as well as independently for the seropositive patients. JCV seropositivity prevalence was estimated overall and for different demographic groups. Univariate logistic regression analysis with anti-JVC seropositivity as the outcome was used to examine the effect of demographic variables on seropositivity. Also, the association between demographic characteristics and JCV AI index was examined using the Kruskal–Wallis test for continuous variables and Fisher's exact test for categorical variables. For all statistical tests, nominal significance was set to *P* < 0.05. Lastly, the seroconversion rate was calculated for natalizumab-treated patients, for the whole duration of their clinical follow-up (from their baseline JCV AI index to the last recorded appointment).

## 3. Results

A total 214 MS patients were tested. The indication for screening was to stratify risk for patients about to be or already treated with natalizumab or other immunomodulatory treatments.

The overall anti-JCV antibody prevalence was 45.8% (95% confidence interval (CI): 37.2%–55.8%). The mean age of all patients was 38.5 years. The percentage of the patients tested that were females was 67.3% (Table 1). Due to incomplete data, four patients were excluded from all age-related statistics and analyses.

**Figure 1:**
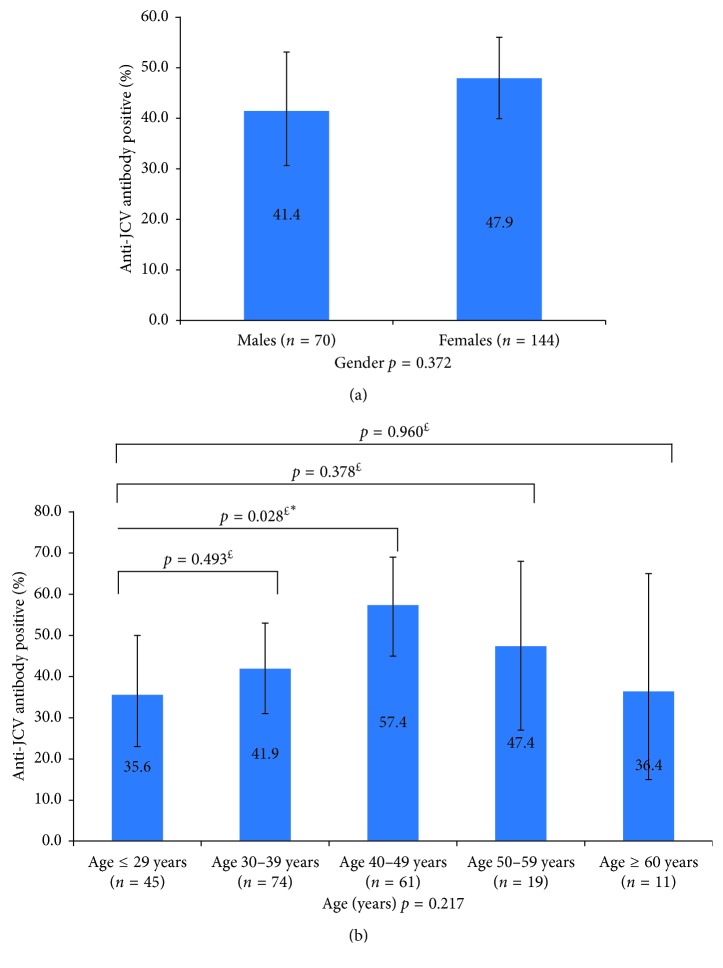
Anti-JCV antibody prevalence by patient demographics: (a) gender and (b) age. The number of patients in each group is shown in the legend of each bar, and vertical bars represent 95% confidence intervals. The ^$^*P* value comparing seroprevalence across all demographic variable categories was determined from univariate logistic regression analysis with anti-JVC seropositivity as the outcome. The ^£^*P* value comparing each age group with the baseline (age ≤ 29) was determined from univariate logistic regression analysis with anti-JVC seropositivity as the outcome. ^*∗*^Statistically significant *P* value.

The prevalence of anti-JCV antibodies did not increase significantly with age when age was treated as a continuous variable (mean age ± SD_negative vs. positive_: 37.7 ± 10.9 vs. 39.6 ± 10.4; *P*=0.202). There was a significant difference, however, when comparing the <29-year age group with the 40–49-year age group (*P*=0.028) (Figure 1). A seropositivity rate of 35.6% was observed in the group of patients <29 years old and 36.4% in the group of patients >60 years old. The highest rate of seropositivity was documented in the group of patients between 40 and 49 years old with 57.4%. The prevalence of anti-JCV antibodies was higher in females than in males (47.9% vs. 41.4%), but this was not statistically significant (Figure 1).

The mean age of the anti-JCV antibody seropositive patients was 39.6 years, and 70.4% were females (Table 2). Forty-nine out of the 98 seropositive patients had an unknown index. Most of the 49 remaining patients (75.5%) had a high antibody index with AI > 1.5 (Table 2); however, no significant association between antibody index and age or gender was found (Table 3).

In our subgroup analysis of 121 natalizumab-treated patients, 14 patients (11.6%) underwent seroconversion from negative to positive during the whole follow-up duration (mean _follow-up_: 3.10 person-years, SD_follow-up_: 1.96 person-years), corresponding to an annual conversion rate of 4.5%. Moreover, in the same period, 5 patients (4.1%) underwent positive to negative seroconversion and another 5 patients (4.1%) displayed alternate seroconversion (Table 4). No patients in our cohort developed PML.

## 4. Discussion

This is the first study in a novel population of MS patients in Cyprus. The overall anti-JCV antibody prevalence in MS patients in Cyprus of 45.8% is one of the lowest recorded in comparison to previous studies assessing the JCV seroprevalence using the STRATIFY JCV assay.

Previous studies reported an association between seropositivity and increasing age and male gender [14, 17, 18]. In those studies, the anti-JCV prevalence differed significantly by country, with no identifiable geographical pattern. Also, disease duration or type and treatment regime did not seem to have any influence on the anti-JCV status. In our study, no significant association between age and gender was found. While the geographical variation in JCV exposure is well documented, the reasons of the low prevalence in Cyprus remain unclear and need to be further investigated. Moreover, our subgroup analysis of natalizumab-treated patients showed an annual 4.5% seroconversion rate which is significantly lower than the reported annual rate in the literature [19, 20].

Our study had several limitations. The number of patients included in our study (*n* = 214) is relatively small to draw statistically significant conclusions, which may explain the lack of association between age and gender which was previously reported in the literature. However, as of 2013, the Cyprus population was 858,000 with a total of 1700 MS patients [21]; therefore, while 214 patients is a small sample in absolute numbers, it is considered representative of the MS population of the whole island.

Our study was also susceptible to selection bias since all our patients were tested for JCV antibodies for risk stratification before or during treatment with (mostly) natalizumab or other immunomodulatory treatments and were not a random sample selected from the whole MS Cypriot cohort. However, we do not believe that the selection bias influenced the results significantly since previous studies failed to reveal an association between disease duration or other clinical characteristics and JCV prevalence [14, 17, 18].

Although our understanding of JCV and PML pathogenesis has grown considerably in the recent years [6], there are still many questions left unanswered and certainly more research is needed. The meticulous study of host genetics and environmental factors in geographical regions with different JCV seroprevalence could enable the identification of potential preventable risk factors associated with JCV infection and consequently with PML.

In summary, the anti-JCV antibody prevalence in MS patients in Cyprus is at the lower end of the spectrum when compared to the prevalence of the global population, but within the previously reported expected range. More research is needed to identify the reasons for the different JCV prevalence in different geographical regions, including the lower prevalence in Cyprus.

## Figures and Tables

**Table 1:** Demographic and clinical characteristics of the cohort (*n* = 214).

Characteristics	Descriptive statistics
Age (years)	
Mean ± SD	38.5 ± 10.7
Median (range)	38 (16–70)
Gender, *N* (%)	
Male	70 (32.7%)
Female	144 (67.3%)
Anti-JCV antibody seropositivity, *N* (%)	
Negative	116 (54.2%)
Positive	98 (45.8%)

SD: standard deviation.

**Table 2:** Clinical characteristics of the anti-JCV antibody seropositive group (*n* = 98).

Characteristics	Descriptive statistics
Age (years)	
Mean ± SD	39.6 ± 10.4
Median (range)	38 (16–70)
Gender, *N* (%)	
Male	29 (29.6%)
Female	69 (70.4%)
JCV_AI_index^*∗*^	
≤0.9	9 (18.4%)
>0.9– ≤ 1.5	3 (6.1%)
>1.5	37 (75.5%)

^*∗*^The antibody index of 49 subjects was not known; therefore, statistics apply to *n* = 49.

**Table 3:** Analysis of the JCV_AI_index of seropositive patients with a JVC AI index (*n* = 49) according to prespecified demographics.

Variables	≤0.9	>0.9– ≤ 1.5	>1.5	*P* value^$^
Mean age^*∗*^	40.8 ± 7.6	39.7 ± 3.5	38.3 ± 10.2	0.587
Age categories^*∗*^				
Age < 30 years	1 (14.3%)	0 (0.0%)	6 (85.7%)	
Age ≥ 30 years	7 (17.9%)	3 (7.7%)	29 (74.4%)	0.999
Gender				
Male	4 (28.6%)	1 (7.1%)	9 (64.3%)	
Female	5 (14.3%)	2 (5.7%)	28 (80.0%)	0.454

^$^ *P* value from the Kruskal–Wallis test for mean age and Fisher's exact test for categorical variables. ^*∗*^Age was missing for 3 patients with a JCV AI index.

**Table 4:** Analysis of seroconversion patterns among patients on natalizumab therapy (*n* = 121).

	No. of patients (%)	Follow-up time (mean ± SD, person-years)	Annual conversion rate	Mean (SD) duration of natalizumab intake (years)
Seroconversion negative to positive	14 (11.6)	3.10 ± 1.96	4.5%	4.5 (2.3)
Seroconversion positive to negative	5 (4.1)	1.63 ± 1.96	1.7%	5.2 (2.2)
Alternate seroconversions	5 (4.1)	Not available^≠^	Not available^≠^	4.3 (2.1)

^≠^Information not available since several events per patient (alternate seroconversions) do not permit assignment of a specific follow-up time, thus making the calculation of an annual conversion rate impossible.

## Data Availability

The data used to support the findings of this study are available from the corresponding author upon request.
